# Calretinin promotes invasiveness and EMT in malignant mesothelioma cells involving the activation of the FAK signaling pathway

**DOI:** 10.18632/oncotarget.26332

**Published:** 2018-11-20

**Authors:** Janine Wörthmüller, Walter Blum, Laszlo Pecze, Valérie Salicio, Beat Schwaller

**Affiliations:** ^1^ Unit of Anatomy, Section of Medicine, University of Fribourg, 1700 Fribourg, Switzerland; ^2^ Genetica AG, 8001 Zurich, Switzerland

**Keywords:** calretinin, malignant mesothelioma, FAK signaling, EMT

## Abstract

Calretinin (CR) is used as a positive marker for human malignant mesothelioma (MM) and is essential for mesothelioma cell growth/survival. Yet, the putative role(s) of CR during MM formation *in vivo*, binding partners or CR’s influence on specific signaling pathways remain unknown. We assessed the effect of CR overexpression in the human MM cell lines MSTO-211H and SPC111. CR overexpression augmented the migration and invasion of MM cells *in vitro*. These effects involved the activation of the focal adhesion kinase (FAK) signaling pathway, since levels of total FAK and phospho-FAK (Tyr^397^) were found up-regulated in these cells. CR was also implicated in controlling epithelial-to-mesenchymal transition (EMT), evidenced by changes of the cell morphology and up-regulation of typical EMT markers. Co-IP experiments revealed FAK as a new binding partner of CR. CR co-localized with FAK at focal adhesion sites; moreover, CR-overexpressing cells displayed enhanced nuclear FAK accumulation and an increased resistance towards the FAK inhibitor VS-6063. Finally, CR downregulation via a lentiviral shRNA against CR (*CALB2*) resulted in a significantly reduced tumor formation *in vivo* in an orthotopic xenograft mouse model based on peritoneal MM cell injection. Our results indicate that CR might be considered as a possible target for MM treatment.

## INTRODUCTION

Malignant mesothelioma (MM) is a highly aggressive neoplasm that arises from mesothelial cells covering the surfaces of the pleura, peritoneum, and pericardium and is prevalently associated with asbestos exposure [1]. The prognosis is extremely poor mostly due to late diagnosis and lack of understanding of its biology and molecular pathogenesis. There is no effective treatment so far; median overall survival following frontline chemotherapy with pemetrexed and cisplatin is approximately 12 months [2], thus early diagnosis is critical, and prolonged survival can be best achieved among patients who are candidates for surgery [1]. The development of MM occurs after a long latency period, suggesting that multiple (genetic) events are required for tumorigenic conversion of mesothelial cells [3]. According to the WHO classification, MM is sub-classified as epithelioid (mostly composed of epithelial-shaped cells), sarcomatoid (composed of spindle-shaped cells), or biphasic (a mixture of both cell types) [4].

The Ca^2+^-binding protein calretinin (CR) serves as an undisputed marker for the diagnosis of MM, in particular of the epithelioid type and the epithelioid parts of the mixed type [5, 6]. CR is essential for MM cell growth/survival *in vitro*, since its downregulation results in decreased cell proliferation and viability [7]. Overexpression of CR in immortalized mesothelial cells protects them from acute asbestos-induced cytotoxicity *in vitro*; thus, an increased survival of asbestos-exposed CR-expressing mesothelial cells may promote and favor MM development [8]. Regulation of CR expression is cell type-specific [9], yet little is known on the mechanisms and effects of CR up-regulation during early stages of mesotheliomagenesis.

Focal adhesion kinase (FAK) is overexpressed in various cancers including MM [10]. Activation of tyrosine kinases is essential in the progression from non-neoplastic mesothelial progenitor cells to mesothelioma [11] and FAK has been often described to promote malignancy by regulating tumorigenesis and metastasis through highly-coordinated signaling networks orchestrating invasion, EMT, angiogenesis and regulation of cancer stem cells [12]. Based on pre-clinical findings, FAK inhibition is of potential therapeutic interest and a number of FAK-directed small molecule inhibitors are currently undergoing clinical trials. EMT, characterized by acquisition of a mesenchymal phenotype, increased migratory and invasive potential, enhanced resistance to apoptosis, and increased production of extracellular matrix (ECM) components, is thought to represent an important step in cancer progression [13, 14]. Besides CR’s well-known function as a Ca^2+^-buffering protein, additional putative CR sensor functions (e.g. binding partners in MM cells) or CR’s possible role(s) during EMT in MM development are largely unknown.

In this study we investigated the role of CR in MM cell lines by evaluating the effects of CR overexpression. Our results demonstrate the essential role of CR in promoting migration, invasion and EMT *in vitro*, likely with an involvement of FAK signaling, since FAK protein levels and the activated phosphorylated form p-FAK (Tyr^397^) were up-regulated in CR-overexpressing cells. We also validated CR downregulation as a promising strategy to impair MM progression *in vivo* in an orthotopic mouse model.

## RESULTS

### CR overexpression promotes migration and invasion *in vitro*

**Figure 1:**
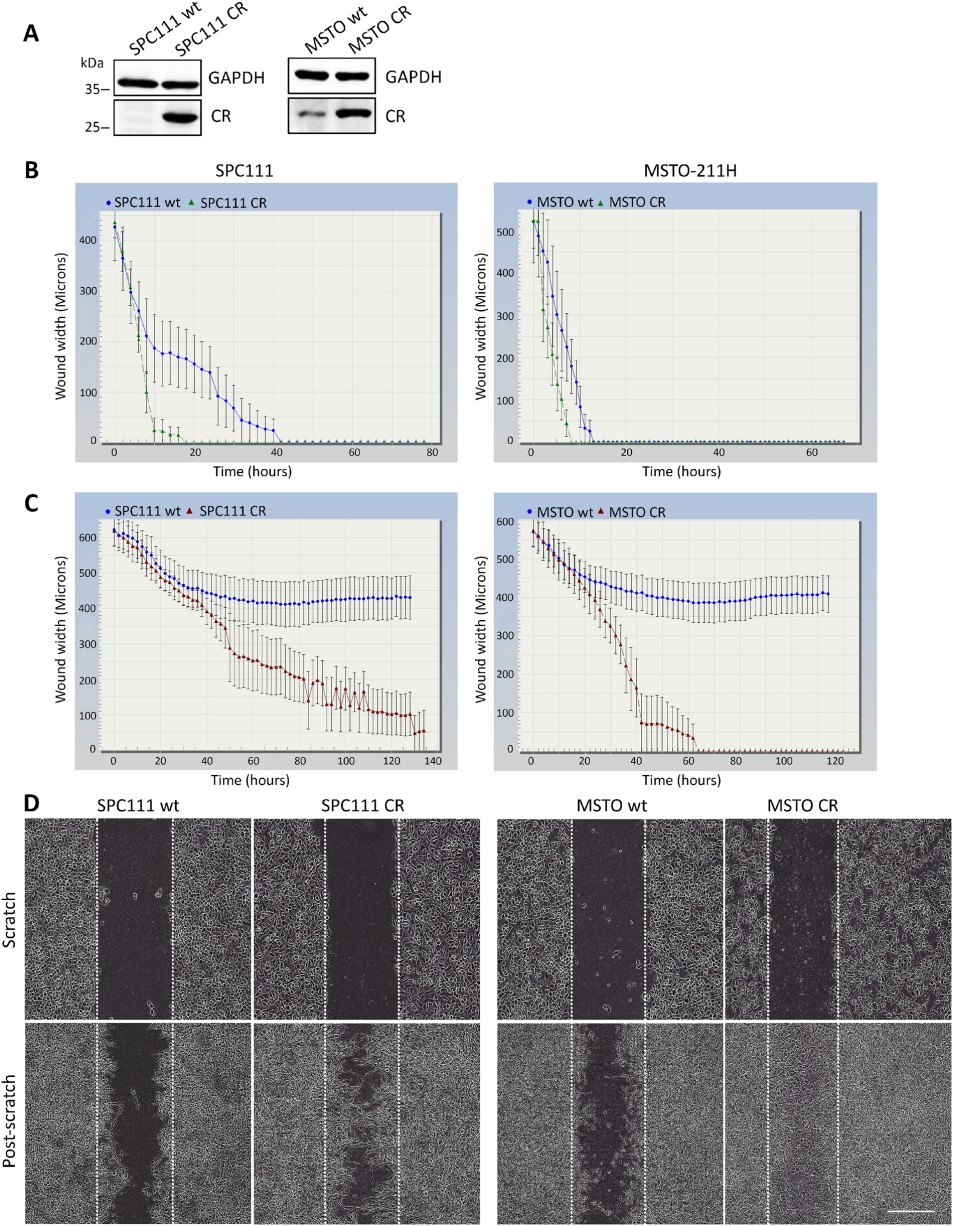
CR overexpression promotes migration and invasion *in vitro* **(A)** Western Blot analysis demonstrating lentiviral-mediated CR overexpression in SPC111 and MSTO-211H cells. GAPDH was used as loading control. **(B)** Comparison of wound closure kinetics between wt (blue) and CR- overexpressing cells (green) shown for SPC111 and MSTO-211H cells monitored with the IncuCyte™ imaging system every 2 h. A faster closing of the scratch is observed for CR-overexpressing cells when compared with wt cells. **(C)** Invasiveness determined by a modified ‘wound healing assay’ with an added matrix barrier component (Matrigel™). Comparison of wound closure kinetics between wt (blue) and CR-overexpressing cells (red). CR-overexpressing SPC111 and MSTO-211H cells completely closed the wound after 90 h and 60 h, respectively. Neither of the wt cells were capable of closing the gap during the observation period (140 h; n = 4 independent experiments). **(D)** Representative examples of time-lapse images taken after confluent monolayers of SPC111 and MSTO-211H (wt and CR-overexpressing cells) were scratched (time point 0 h), and after 90 h and 60 h (post-scratch), respectively. Scale bar: 400 μm.

The effect of CR overexpression was investigated in SPC111 (low endogenous CR levels) and MSTO-211H cells (medium-to-high endogenous CR levels), both MM cell lines from the biphasic subtype. Strong lentivirus-mediated CR overexpression was evident in both lines (Figure 1A). No apparent differences in the morphology of the cells were observed after short-term CR overexpression (data not shown). Although CR overexpression tended to initially increase cell proliferation (generally observed between passages 2-4), at later passages (>5) no differences were observed when compared with the parental (wt) cells (Supplementary Figure 1A). Analyses of CR levels at early (<10) and late passages (>30) showed no significant changes in CR protein levels (Supplementary Figure 1B); if anything, a slight (insignificant) increase in CR levels was observed at later passages. Thus, lentiviral-mediated CR expression resulted in stable and permanently elevated CR expression levels of this protein in SPC111-CR and MSTO-CR cells. Since local invasion is the characteristic clinical feature of MM, and mesothelin, another typical marker of mesothelioma, increases migration and invasion in MM cells [15], the effect of CR overexpression on cell migration/proliferation and invasion was investigated. In a scratch wound assay, the closing of a gap of approximately 1 mm by cell proliferation and migration was monitored. Video/microscope-based scratch wound assays represent a robust technique to evaluate cell migration and invasion, presenting some advantages over the typical Boyden chamber assay [16]. Time-lapse experiments revealed an accelerated and complete closing in both CR-overexpressing lines compared to the wt cells (Figure 1B). The effect was more pronounced in SPC111 cells. As proliferation rates were identical in wt and CR-overexpressing cells (Supplementary Figure 1A), differences in the wound closure times between CR-overexpressing and wt cells were attributed to differences in the migration capacity of the cells. On average, the closure of the wound was completed after 18 h with SPC111-CR cells, while SPC111-wt cells showed a significant delay, closing the wound after 38 h (p ≤ 0.0001) (Supplementary Figure 1C). For MSTO-211H cells, the corresponding wound closure times of CR-expressing and wt cells were 10 h and 16 h, respectively (p ≤ 0.05) (Supplementary Figure 1C). Invasion was assessed with a modified wound-healing assay, where the gap was filled and cells were covered with a basement membrane matrix (Matrigel; 1 mg/ml) substrate that mimics cell-matrix interactions in the tumor microenvironment. Both MM cell lines overexpressing CR were clearly more invasive than the corresponding wt cells (Figure 1C, 1D). SPC111-CR closed the wound almost completely after 90 h, while in the case of MSTO-CR cells the closure of the wound was already complete after 60 h (see Supplementary Movies 1-4). Neither of the wt cells were capable of completely invading the gap space at any time point (measured up to 140 h). Invasion was additionally measured with the Boyden chamber assay (Supplementary Figure 2). CR-expressing SPC111 cells showed significantly increased invasive potential compared to the corresponding wt cells, thus confirming the results obtained with the scratch assay (Figure 1). Of note, cell migration/proliferation was not different between SPC111 wt and SPC111-CR cells determined in this assay (Supplementary Figure 2).

### CR overexpression leads to FAK up-regulation and altered FAK localization

**Figure 2:**
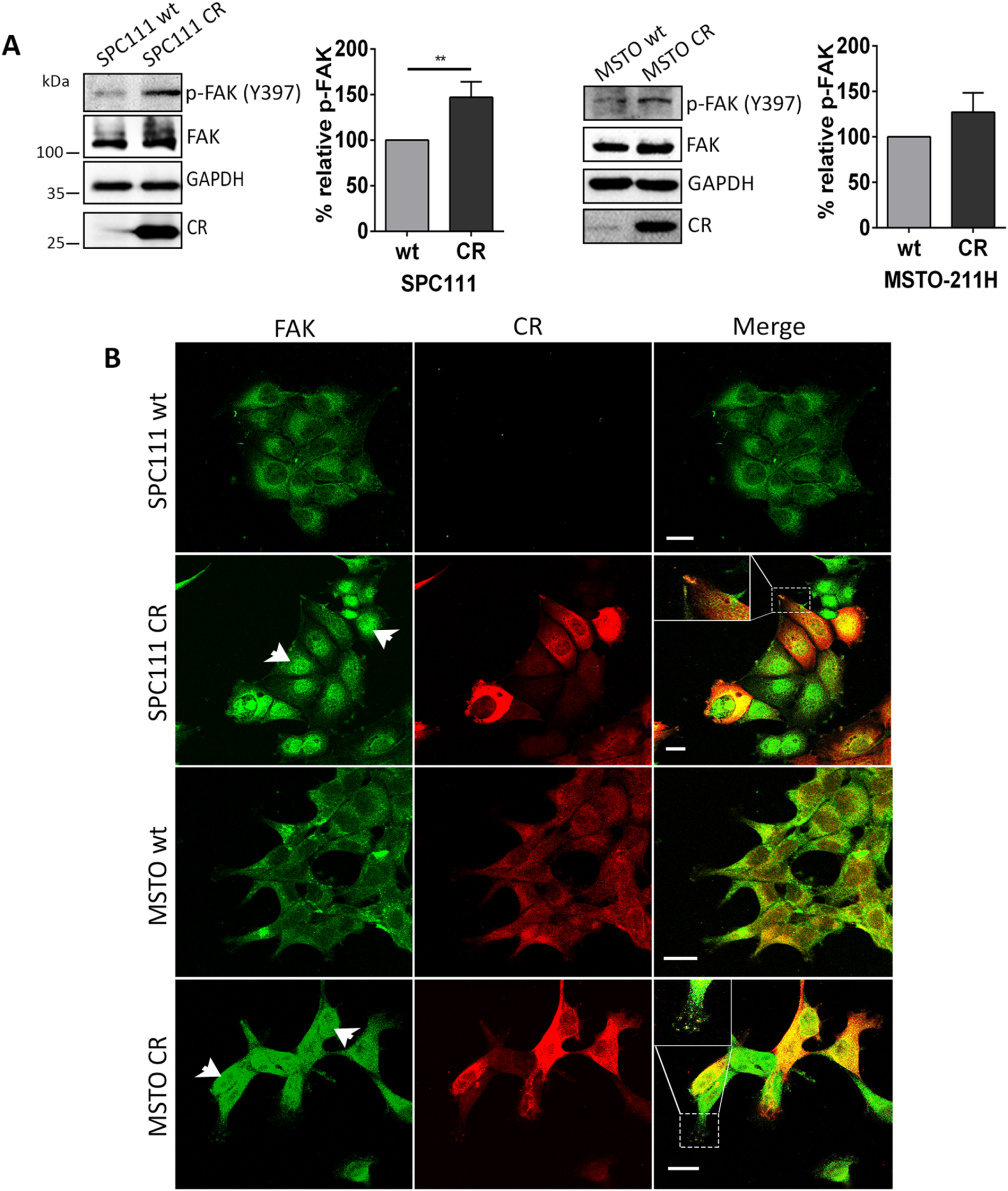
CR overexpression leads to FAK up-regulation and altered FAK localization **(A)** Western Blot analysis of CR-overexpressing and wt MM cells revealed up-regulation of total FAK and p-FAK (Tyr^397^) in SPC111 and MSTO-211H cells. Protein size markers are shown in the first Western Blot of the figure. GAPDH was used as loading control. Quantification of Western Blot signals for p-FAK (Tyr^397^) is shown. p-FAK (Tyr^397^) signals were increased by ≈50% in SPC111-CR and ≈40% in MSTO-CR with respect to the corresponding wt cells (^**^p ≤ 0.01; n = 3 independent experiments). **(B)** Representative confocal images from fixed cells stained for FAK (green) and CR (red) in wt and CR-overexpressing cells. In both cell lines CR co-localized with FAK (represented by yellow color in the merged images) in some parts of the cytosolic region and at the leading edge of cells forming punctate-like patterns (focal adhesions) along the plasma membrane. An enlarged image is shown on the top left of the merged image. CR-overexpressing cells exhibit nuclear FAK accumulation (arrowheads). Scale bar: 20 μm.

Among proteins favoring tumor invasion, elevated FAK expression is associated with increased tumor invasiveness in several malignancies [17, 18]. Total FAK levels were upregulated in CR-overexpressing SPC111 and MSTO-211H cells when compared with the corresponding wt cells (Figure 2A). Additionally, signals for phospho-FAK modified at the major autophosphorylation site Tyr^397^ were stronger in CR-overexpressing cells. Quantification of p-FAK (Tyr^397^) Western Blot levels revealed a significant increase of approximately 50% in SPC111-CR cells with respect to the corresponding wt cells (p ≤ 0.01). A trend towards an increase of p-FAK (Tyr^397^) levels was also observed in the case of MSTO-CR cells (increase of 27±12%; n.s.). Analyses of the ratios p-FAK (Tyr^397^)/FAK revealed a significant increase (1.314±0.001 (normalized to wt); p ≤ 0.0001) in MSTO-CR cells compared to wt cells. In SPC111 cells, this ratio was also slightly increased in CR-overexpressing SPC111 cells (1.076±0.071; n.s.); the reason for not reaching significance in SPC111 cells is resulting from a nearly paralleled increase of both, p-FAK (Tyr^397^) and total FAK. Total FAK levels were also clearly increased in epithelioid MM cells (ZL5 and ZL55) and to a lesser extent in the biphasic SPC212 and sarcomatoid ZL34 MM cells (Supplementary Figure 3A). However, in the latter two lines, signals for p-FAK (Tyr^397^) were strongly increased in the CR-overexpressing cells (Supplementary Figure 3A). Besides increasing total FAK and p-FAK (Tyr^397^) levels, CR overexpression also affected FAK’s intracellular localization. In both MSTO-211H wt and -CR cells, CR immunofluorescence was distributed rather homogenous; nuclear CR staining was somewhat increased in MSTO-CR cells (Figure 2B). While CR levels in SPC111 wt cells were below the detection threshold, immunostaining intensity of CR-overexpressing cells was rather heterogeneous, likely the result of different copy numbers and integration sites of the transgene (Figure 2B). FAK staining was relatively weak and more confined to perinuclear regions of SPC111 and MSTO-211H wt cells. In both lines with elevated CR levels, CR and FAK showed a strong co-localization at the leading edge of the cells forming punctate-like patterns along the plasma membrane typical of focal adhesions. A similar co-localization was also observed in other CR-overexpressing MM lines (e.g. ZL55-CR and ZL5-CR; Supplementary Figure 3B). Unexpectedly, in both CR-overexpressing MM lines, nuclear FAK immunofluorescence was significantly increased, particularly evident in SPC111-CR and to a lesser extent in MSTO-CR cells (Figure 2B).

### Nuclear FAK levels are increased in CR-overexpressing cells. Co-IP experiments reveal a direct interaction between CR and FAK

**Figure 3:**
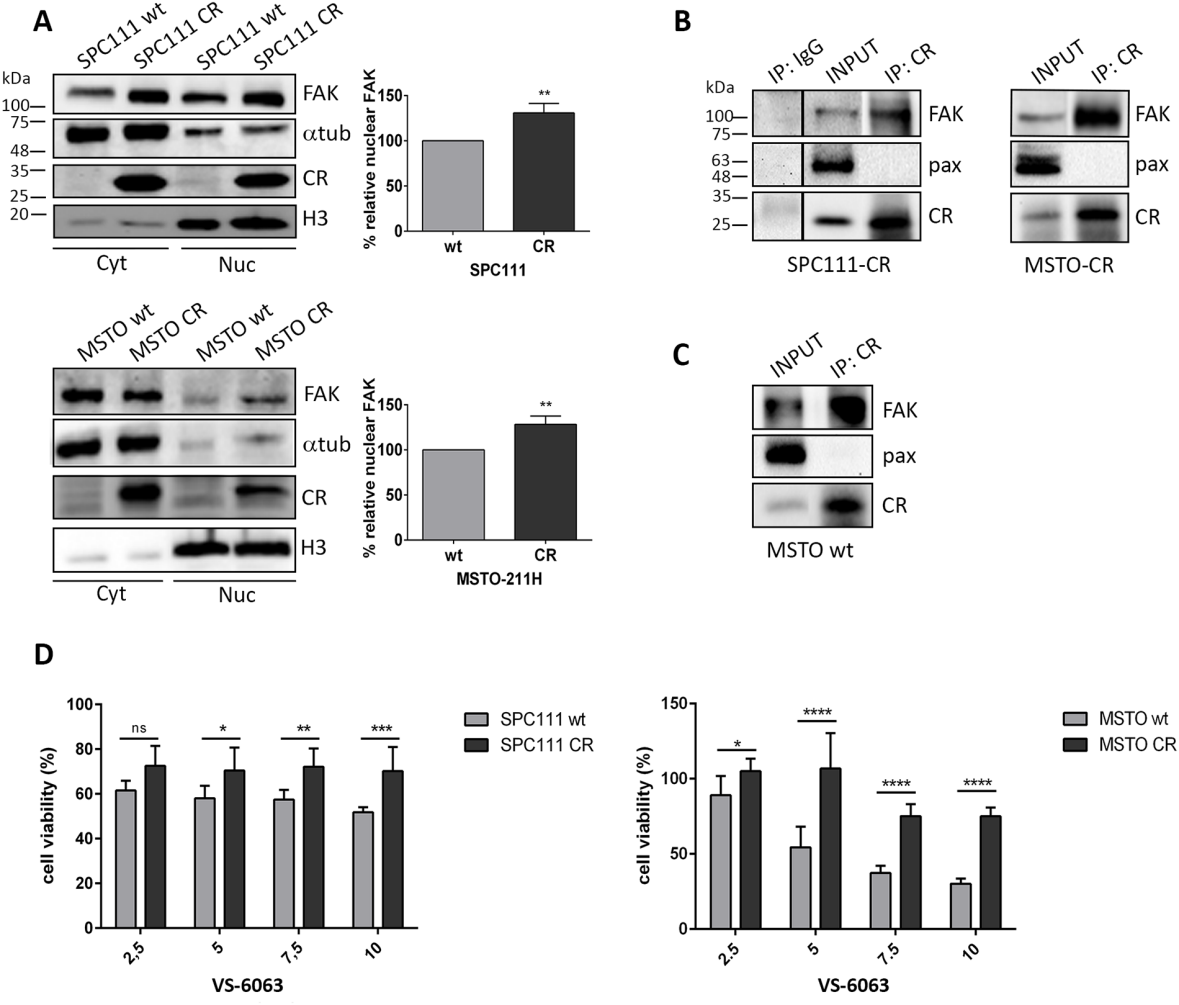
CR-expressing cells show increased nuclear FAK levels and an increased resistance towards the FAK inhibitor VS-6063; Co-IP experiments reveal an interaction between CR and FAK **(A)** Western Blot analysis of cytosolic and nuclear protein extracts demonstrated an increase of nuclear FAK levels in both CR-overexpressing MM cell lines when compared with wt cells. Protein size markers are shown in the first Western Blot of the figure. α-Tubulin and Histone H3 antibodies were used to demonstrate enrichment of these proteins in cytoplasmic and nuclear protein fractions, respectively. In the graphs the relative percentage of nuclear FAK expression levels is represented (^**^p ≤ 0.01; n = 3 independent experiments). **(B)** Co-IP experiments with cellular lysates from SPC111-CR, MSTO-CR and **(C)** MSTO wt cells. FAK/CR complexes were co-immunoprecipitated with CR antibodies. A sample collected prior to IP is shown as INPUT. A normal rabbit polyclonal IgG was used as a negative IP control. Western Blot analysis was performed using anti-CR, anti-FAK and anti-paxillin (negative control for CR-binding) antibodies. Representative blots from 3 independent experiments. **(D)** Cell viability measured with the MTT assay (48 h for SPC111 and 72 h for MSTO-211H cells) after treatment with the FAK inhibitor VS-6063 at different concentrations ranging from 2.5 μM to 10 μM. CR-overexpressing cells of both cell lines showed an increased resistance towards the inhibitor when compared with wt cells (n = 3 independent experiments; asterisks represent ^*^ p ≤ 0.05, ^**^p ≤ 0.01, ^***^p ≤ 0.001, ^****^p ≤ 0.0001, respectively).

Western Blots of nuclear extracts from CR-overexpressing MM cells showed stronger FAK signals than extracts from the corresponding wt cells (Figure 3A); the increase of approximately 30% was significant in SPC111 and MSTO-211H cells (p ≤ 0.01). The increase was also evident in cells co-stained with DAPI and FAK resulting in cyan co-staining (Supplementary Figure 4). Since FAK and CR co-localized at focal adhesions and moreover in the nuclei of some CR-overexpressing MM cells (Figure 2B), we investigated putative interactions between FAK and CR by co-immunoprecipitation experiments. FAK was undoubtedly pulled down by CR antibodies indicative of direct physical interaction (Figure 3B). Additionally, FAK was co-immunoprecipitated with CR in MSTO wt cells, which endogenously express relatively high CR levels, thus precluding that the interaction might be an artifact taking place only in genetically modified (CR-overexpressing) cells (Figure 3C). The complementary experiment, CR being pulled down by FAK antibodies was negative using two different FAK antibodies (data not shown). This failure was attributed to FAK antibodies binding to an epitope in FAK overlapping with the CR interaction site and thus sterically hindering the interaction with CR.

### Cells overexpressing CR exhibit an increased resistance towards the FAK inhibitor VS-6063

Since FAK inhibitors decrease tumor growth and metastasis in preclinical models and in initial clinical trials in cancer patients [19, 20] and FAK expression/activity was increased in CR-overexpressing MM cells, cells were exposed to the FAK inhibitor VS-6063. When compared to the respective wt controls, CR-overexpressing cells of both cell lines were more resistant at higher concentrations (≥ 5 μM) of the inhibitor (Figure 3D), evidenced by the increase in viability after 48 h (SPC111) and 72 h (MSTO-211H) of treatment. Differences became most evident at the highest inhibitor concentration (10 μM). These findings are in support of CR overexpression in MM cells leading to enhanced FAK signaling, subsequently requiring higher VS-6063 concentrations to decrease cell viability.

### CR overexpression induces EMT

**Figure 4:**
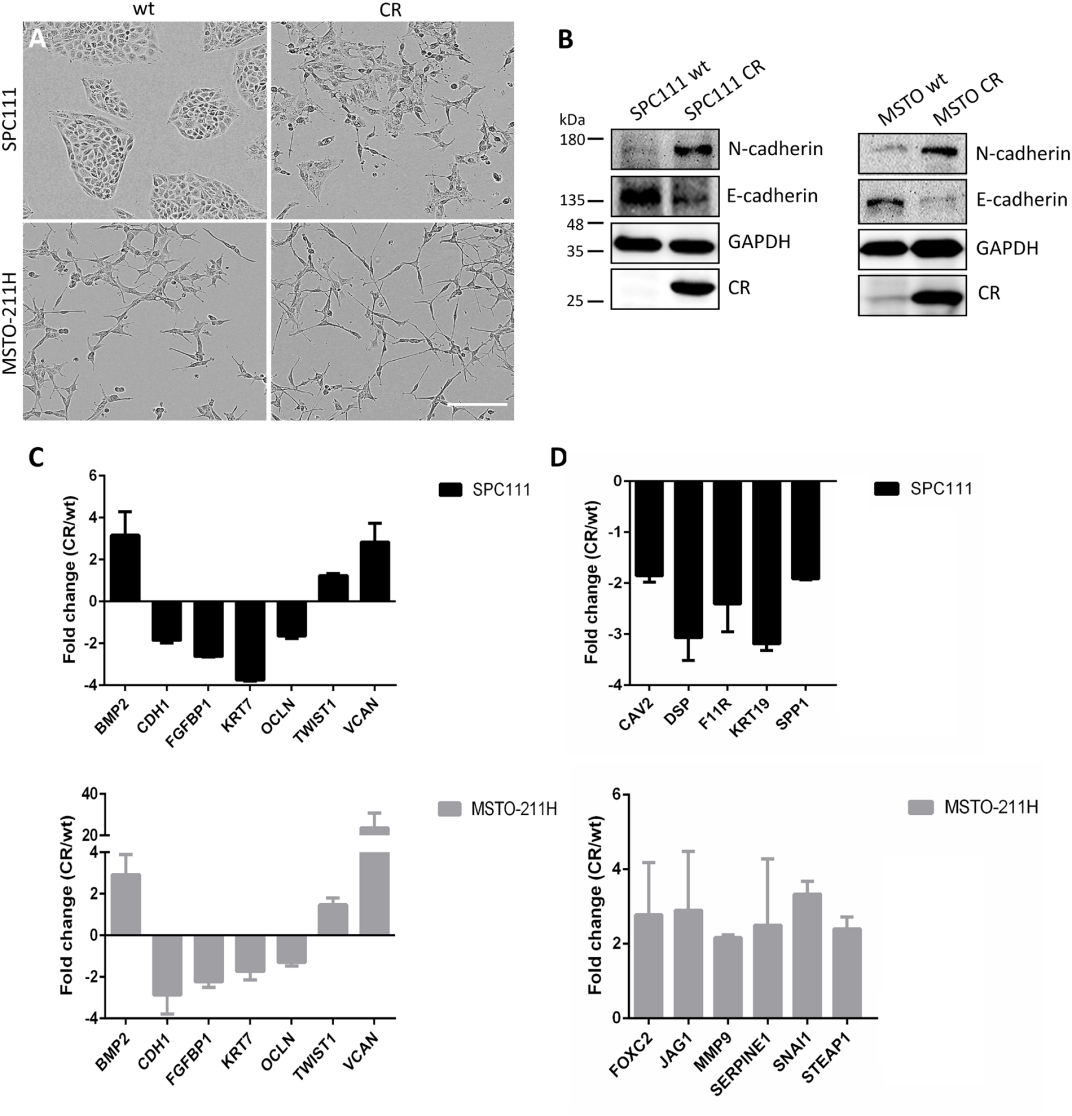
CR overexpression in MM cells induces EMT **(A)** Representative images of wt and long-term CR-overexpressing MM cells. SPC111-CR cells showed loss of the typical clustered morphology and cell scattering. MSTO-CR cells exhibited an increased spindle-like phenotype. Scale bar: 400 μm. **(B)** Western Blot analysis of the epithelial marker E-cadherin and the mesenchymal marker N-cadherin in wt and CR-overexpressing cells. CR overexpression induced a reduction of E-cadherin and an increase in N-cadherin when compared with wt cells. GAPDH was used as loading control. **(C)** Relative fold-change of transcript expression determined by an EMT pathway-specific PCR array (mean ± SD from 2-3 independent experiments). Transcripts similarly up- and downregulated in both SPC111-CR (black) and MSTO-211H-CR (gray) in comparison to their wt cells are shown in **(C)**; differently expressed transcripts in either MM cell line are shown in **(D)**

Cell invasion is considered a key step in metastasis resulting from various factors and altered oncogenic signaling pathways, which overlap with EMT-inducing pathways [21], often with an involvement of FAK [22]. Since transient CR expression in the mesenchyme occurs during normal embryonic development of the mouse lung [23], and CR expression is increased in mice subjected to bleomycin treatment resulting in EMT [24], we investigated the effect of long-term CR overexpression in MM cells on EMT. At the morphological level, CR overexpression in SPC111 cells resulted in a change from well-defined compact clusters (wt), to more loosely attached and often dispersed cells (Figure 4A). Likewise, MSTO-CR cells showed a loss in the connection of the cells with a change towards a more spindle-like morphology when compared with wt cells. The prototypical epithelioid marker E-cadherin, its downregulation considered as a hallmark of EMT [25], was found decreased at the protein level in both CR-overexpressing MM lines, while N-cadherin, a mesenchymal marker that increases during EMT, was upregulated (Figure 4B). A specific EMT pathway PCR array containing 84 key genes allowed further investigation of the role of CR on EMT (Figure 4C, 4D). Commonly downregulated transcripts in CR-overexpressing MM cells included typical epithelial markers as *CDH1* (E-cadherin); *KRT7*, a cytokeratin associated with a basal phenotype [13] and *OCLN* (occludin), which controls epithelial tight junctions. Also *FGFBP1* encoding Fibroblast Growth Factor Binding Protein 1, a protein implicated in EMT, was decreased. Genes up-regulated in both cell lines were *BMP2*, a bone morphogenetic protein, known to promote motility and invasion in several cancers [25], *VCAN* (Versican), a chondroitin sulfate proteoglycan that modulates cell proliferation, differentiation and adhesion [26] and the EMT marker *TWIST1*. Genes up-regulated more than 2-fold in MSTO-CR cells included *FOXC2* (forkhead box C2) and *JAG1* (protein-jagged1), both involved in the Notch signaling pathway and known to be increased during EMT [13, 25], *MMP9* (Matrix MetalloPeptidase 9), an important extracellular matrix-degrading enzyme that enables invasion [25], as well as *SERPINE1* and *STEAP1*, genes involved in ECM processes and cell adhesion. Also signals for *SNAI1* (encoding the transcription factor Snail), which controls tumor growth and stemness and is considered as typical EMT marker, were augmented in MSTO-CR cells. Transcripts downregulated in SPC111-CR cells included *CAV2* encoding the potent tumor suppressor caveolin-2, *SPP1* (osteopontin) and cytokeratin 19 (*KRT19*), as well as *DSP* and *F11R*, genes implicated in the organization of desmosomes and tight junctions, respectively [13, 25]. Other up- and/or downregulated genes are summarized in Table 1. Thus, increased invasiveness of CR-overexpressing MM cells is the likely consequence of an EMT process.

**Table 1:** Up- and downregulated genes associated with CR overexpression in MM cells

SPC111			
Gene	Gene full name	Related gene function^*^	Fold change
ERBB3	v-erb b2 erythroblast leukemia viral oncogene homolog 3	Differentiation & development; Cell growth & proliferation; Extracellular matrix & cell adhesion; Receptor tyrosine kinase signaling pathway.	-1.5
ESR1	Estrogen receptor 1	Transcription factor; Estrogen-receptor signaling pathway.	-2
IGFBP4	Insulin-like growth factor binding protein 4	Up-regulated during EMT; Cell growth & proliferation.	2.5
MAP1B	Microtubule Associated Protein 1B	Cytoskeleton.	1.6

MSTO-211H			
Gene	Gene full name	Related gene function	Fold change
ERBB3	v-erb b2 erythroblast leukemia viral oncogene homolog 3	Differentiation & development; Cell growth & proliferation; Extracellular matrix & cell adhesion; Receptor tyrosine kinase signaling pathway.	-1.5
CAMK2N1	Ca^2+^/calmodulin-dependent protein kinase II inhibitor 1	Up-regulated during EMT.	2
DSC2	Desmocollin 2	Extracellular matrix & cell adhesion.	-1.7
IL1RN	Interleukin 1 Receptor Antagonist	Downregulated during EMT.	-1.6
MSTR1	Macrophage stimulating 1 receptor	Downregulated during EMT; Migration & motility; Cell differentiation & development; Cell growth & proliferation.	-2
PDGFRB	Platelet-derived growth factor receptor, beta polypeptide	Cell growth & proliferation; Migration & motility; Receptor tyrosine kinase signaling pathway.	-1.6
TGFB1	Transforming growth factor, beta 1	Morphogenesis; Cell growth & proliferation; Migration & motility; TGFβ/BMP signaling pathways.	1.5
TMEFF1	Transmembrane protein with EF-like and two follistatin-like domains	Up-regulated during EMT; Differentiation & development.	1.6
TMEM132A	Transmembrane protein 132A	Up-regulated during EMT.	1.7

^*^details shown under: https://www.qiagen.com/ch/shop/pcr/primer-sets/rt2-profiler-pcr-arrays/?catno=PAHS-090Z#geneglobe

### CR downregulation *in vivo* impairs tumor progression in a MM orthotopic xenograft mouse model

**Figure 5:**
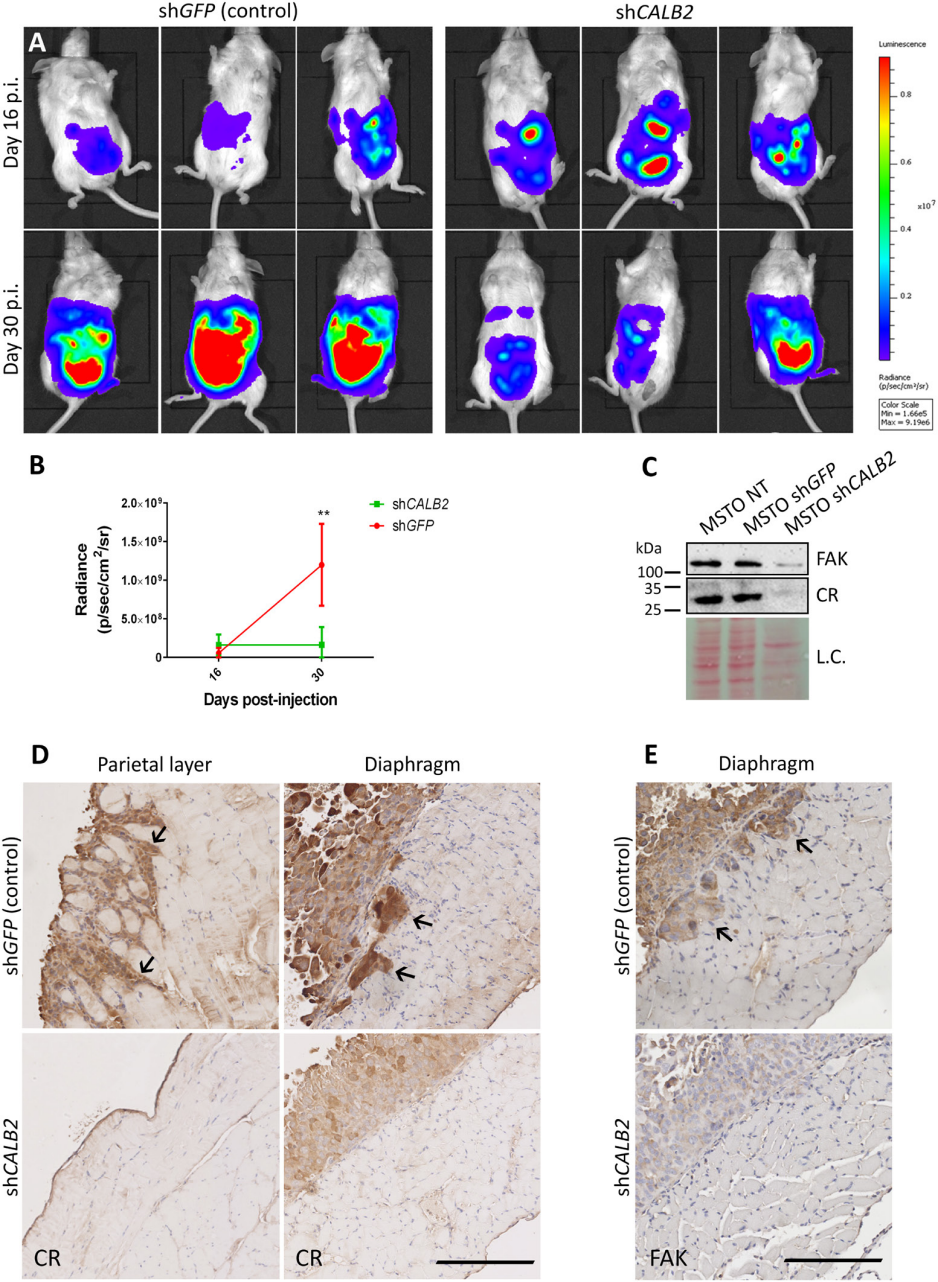
CR downregulation *in vivo* impairs tumor progression in a MM orthotopic xenograft mouse model **(A)** Representative bioluminescence images of tumor burden in NSG mice inoculated with MSTO-211H-Rluc cells pre-treated with a lentiviral vector containing either an shRNA against *GFP* (control group) or against *CALB2* (test group). Mice were scanned at days 16 and 30 p.i. At day 30 p.i., mice treated with shCALB2 showed a decrease in the tumor growth when compared with the control group (treated with shGFP). **(B)** Quantitative analyses of data shown in A. Mean bioluminescent signals (photons/s/cm^2^/sr) obtained from both groups. At day 30 p.i., the shCALB2 group showed a significant reduction (^**^p ≤ 0.01) in the tumor burden when compared with the control group. **(C)** Western Blot analysis demonstrated CR downregulation *in vitro* after 3 days of shCALB2 but not shGFP transduction in MSTO-211H wt cells. In parallel, a decrease of total FAK protein levels after shCALB2 treatment was observed. Ponceau Red staining intensity was used as loading control (L.C.). **(D)** Immunohistochemical staining of CR in the peritoneal parietal layer and diaphragm and **E**. of FAK in the diaphragm from representative sections taken from both groups at day 30 p.i. Arrows denote CR-positive and FAK-positive cells infiltrating the skeletal muscles of the parietal wall and/or the diaphragm present only in the shGFP group. Scale bar: 250 μm.

Since CR downregulation by *CALB2* shRNAs decreases cell growth and viability in MM cells *in vitro* [7], we investigated the effect of CR downregulation within an appropriate tumor microenvironment in an orthotopic mouse model. Animals were randomized into two groups and MSTO-211H-Rluc cells (1.5x10^6^) transduced 24 h earlier with a lentiviral vector containing an shRNA against GFP (control group) or against CR (test group) were injected intraperitoneally. As reported previously, bioluminescent imaging (BLI) in MM was used to non-invasively quantify tumor burden and progression [15]. At day 16 post-injection (p.i.) both mouse groups presented an equivalent BLI signal. At day 30 p.i., tumors had significantly grown in the control shGFP group, but remained unchanged in the shCALB2 group (Figure 5A, 5B). Constitutive downregulation of CR in MSTO-211H (wt) cells *in vitro* resulted in a reduction of ≈90% at the protein level and a similar decrease in total FAK levels (Figure 5C). Tissue samples from MSTO-211H-injected mice (both shGFP and shCALB2) were histologically examined. In mice exposed to shGFP-treated (control) MSTO-211H cells, strongly stained CR-ir cells infiltrating the skeletal muscle of the diaphragm and the parietal peritoneal wall were observed (Figure 5D, upper panels) indicative of high invasiveness. The injection of shCALB2-treated cells did not result in significant changes of the mesothelium of the parietal wall; the few adherent CR-ir MSTO-211H cells mostly formed a single cell layer. On the surface of the peritoneal side of the diaphragm, a thickening of the mesothelium by proliferating MSTO-211H cells was evident; however, no cell infiltration of the skeletal muscle layer was observed in any of the shCALB2-treated mice (Figure 5D, lower panels). Additionally, in mice injected with shCALB2-treated MSTO-211H cells, FAK staining of the tumor cells mostly confined to the thickened tunica serosa was weaker (Figure 5E, lower panel) than in mice injected with the shGFP-MSTO-211H cells (Figure 5E, upper panel). CR-expressing tumor cells infiltrating the muscle tissue were also stronger stained for FAK, in line with the *in vitro* results shown in Figure 5C. Thus, MSTO-211H cells with higher CR and subsequently higher FAK levels showed a higher propensity for tumor cell infiltration in the muscle tissue underneath the tunica serosa.

## DISCUSSION

Mechanisms implicated in the transformation of mesothelial cells to MM are still poorly understood. Pathways dysregulated in MM are related to proliferation, differentiation, migration and invasion, survival, apoptosis, cell cycle control and metabolism, often accompanied by mutations in cell cycle control (*CDKN2A*) and tumor suppressor genes (*BAP1* and *NF2*) [27]. However, the interactions among all these pathways and/or altered genes, as well as their exact role during MM pathogenesis remain unclear. Differential protein expression profiles between benign control and crocidolite-treated Met-5A mesothelial cells revealed activation of pathways related to DNA damage repair and cell cycle regulation and comparison between Met-5A and MM cells (NCI-H28) showed increased activation of the EGFR/ERK and PI3K/AKT pathways [28].

CR is transiently expressed in lung mesenchyme and developing mesothelial cells [29], undetectable in the normal mesothelium, however reappearing in reactive mesothelial cells *in vivo* and immortalized mesothelial cells *in vitro*. These findings together with the presence of CR in epithelioid MM and the epithelioid part of biphasic MM [5, 6] are indicative of an involvement of CR in the initial processes of carcinogenesis. Increasing SV40 early region gene products (TAg, tAg) cause CR up-regulation in Met-5A cells resulting in increased resistance towards acute asbestos-induced cytotoxicity *in vitro*; the same holds true, when directly overexpressing CR [8]. The higher survival of asbestos-exposed high CR-expressing Met-5A cells is mainly mediated through the PI3K/AKT signaling pathway assumed to promote and favor MM development. CR regulation is different in neurons and non-neuronal cell types; in CR-positive colon cancer cells, CR expression is downregulated by butyrate [9] mostly functioning as a histone deacetylase (HDAC) inhibitor [30, 31]. In human MM cells, NRF-1 and E2F2 act as specific trans-activating factors binding to the *CALB2* promoter (−161/+80bp) [32]. In the promoter region of another MM marker gene *MSLN* encoding mesothelin, a “cancer-specific” element driving mesothelin overexpression in cancers was discovered [33]. When introducing this *MSLN* promoter element upstream of the large T antigen gene in the transgenic MexTAg mouse model [34], the promoter becomes active in asbestos-exposed mesothelial cells, leading to asbestos-induced mesotheliomas. Further studies to identify possible asbestos-induced “cancer-specific” promoter elements that are able to up-regulate CR in “primed” and/or early transformed MM cells are needed.

A pathway often dysregulated in many cancers is the FAK signaling pathway. FAK clusters at focal adhesion structures and regulates cancer-associated processes, including adhesion, migration and invasion [35]. Here we show that CR overexpression in MM cells increased total FAK levels and more importantly, FAK tyrosine phosphorylation at the major FAK auto-phosphorylation site Tyr^397^. Levels of p-FAK (Tyr^397^) are considered as proxy measure for augmented FAK signaling [22]. In line, MSTO-CR and SPC111-CR cells with higher p-FAK levels showed higher resistance to the FAK inhibitor VS-6063, likely the result of increased FAK signaling in CR-overexpressing cells. Moreover, FAK expression and key activation phosphorylation sites Tyr^397^ and Tyr^576^ are often elevated in invasive human cancers [12].

The mechanism how elevated CR levels increase FAK expression and/or FAK phosphorylation in MM cells is currently unknown, yet regulation of FAK is an area of intensive investigation. The expression and/or function of FAK “is tightly controlled via modulation of gene expression, competing alternatively spliced forms, non-coding RNAs, and proteins that directly or indirectly affect kinase activation or protein stability” (reviewed in [36]). Some of the above processes of FAK regulation might also be operational in the case of CR. CR functions as a transcriptional co-regulator by binding to the promoter of *SEPT7* coding for the protein septin 7 [37]. A similar mechanism might be envisaged also for the *PTK2* (FAK) promoter. The *CALB2* gene is also subject to alternative splicing producing among others also the transcript *CALB2b*, likely acting as a long non-coding RNA [38]. Finally the interaction of CR with FAK at adhesion sites and/or in the nucleus as reported in this study might result in a protein complex (possibly together with additional proteins) involved in the regulation of FAK expression. These different possibilities should be investigated in further studies.

In CR-overexpressing MM cells, CR not only co-localized with FAK at focal adhesions sites, but both proteins physically interacted, evidenced by Co-IP experiments. Besides CR’s function as a Ca^2+^ buffer, direct binding partners including the pore forming unit of the P/Q type Ca^2+^ channel and a mutant form of huntingtin have also been identified [39, 40]; for more details about CR’s sensor functions, see [41]. CR and paxillin, a focal adhesion-associated protein that functions downstream of FAK as an adaptor protein recruiting diverse cytoskeletal and signaling proteins into an adhesion complex [42], share a common motif, the so-called LD domain [43]. Paxillin’s LD2 and LD4 motifs function as FAK-binding sites [44, 45] and show high homology to the C-terminal part of CR’s EF-hand 5 (Supplementary Figure 5). Thus, we hypothesize that CR might interact with its LD domain to FAK, presumably competing with paxillin binding and modifying FAK-paxillin signaling.

CR overexpression also affected FAK localization by targeting FAK to the nucleus. Besides the canonical roles for FAK, the role of nuclear FAK in regulating cancer cell proliferation and motility is slowly emerging [22]. FAK contains putative nuclear localization sequences (NLS) within the F2 lobe of its FERM domain and can localize to the nucleus, e.g. upon cellular stress, where it binds to p53 [46]. Reported nuclear FAK functions include transcriptional regulation of inflammatory cytokines and chemokines that promote an immuno-suppressive, pro-tumorigenic microenvironment [47]. Due to the well-established role of FAK promoting tumor malignancy, pharmacological blocking of FAK signaling by compounds such as VS-6063 is currently investigated in clinical trials. The increased resistance of CR-overexpressing MM cells to VS-6063 observed in this study is thus assumed to be the result of CR-mediated up-regulation of FAK signaling.

CR overexpression also increased migration and more important invasion of MM cells, likely associated with this enhanced FAK signaling activity. FAK signaling-mediated effects on cell migration and driving cell invasion are well characterized (reviewed in [12]). Tumor invasion by the penetration of cancer cells through the ECM and into neighboring tissue, requires combined effects of enhanced cell motility and alterations in the dynamics of focal adhesions, together with proteolytic degradation of the matrix [48, 49], e.g. by matrix metallopeptidases such as MMP9, shown to be increased in MSTO-CR cells. In support of a role of FAK in increasing the invasiveness, strongly stained FAK-immunoreactive cells were found infiltrating the diaphragm of mice injected with the control shGFP-MSTO cells. Increased FAK is also an essential mediator of EMT [22]. In both MM cell lines, CR-mediated up- and downregulation of specific EMT markers indicating that CR appears to control EMT through multiple pathways and downstream targets. This includes the up-regulation of transcription factors such as twist family bHLH transcription factor 1 (*TWIST1*) and snail family transcriptional repressor 1 (*SNAI1*), as well as an increase of *BMP2*, known to promote motility and invasion. While the effect of CR overexpression on motility was rather mild, the effects on invasion were more striking. Neither of the wt MM cell lines was capable of closing the gap in the *in vitro* invasion assay, while CR-overexpressing ones completely recolonized the Matrigel-filled gap. In support, *in vivo* invasiveness of MSTO-211H cells was strongly decreased upon CR downregulation. Other differentially expressed genes included genes related with proliferation (*VCAN*, *FOXC2*, *JAG1*). CR-mediated downregulation of genes associated with an epithelial phenotype included E-cadherin (*CDH1*, also shown at the protein level, Figure 4B), cytokeratins (*KRT7*, *KRT19*), and occludin (*OCLN*). In line with CR-induced EMT, CR-overexpressing cells were only loosely connected to each other and most had a spindle-shaped morphology.

CR up-regulation at early stages of MM pathogenesis, possibly mediated by asbestos-induced specific transcriptional programs, leads to the activation and up-regulation of the FAK signaling pathway, inducer of migration, invasion and EMT. Decreased tumor growth and invasiveness *in vivo* of MM cells with blocked CR expression reveals CR downregulation as a novel strategy to treat MM, likely in combination with the existing ones (cisplatin, pemetrexed). Reduced CR levels are expected to additionally increase the susceptibility to FAK inhibitors, drugs already being tested in clinical trials.

## MATERIALS AND METHODS

### Cell culture

Human mesothelioma (MSTO-211H), HeLa and HEK293T cells were obtained from the American Type Cell Collection (ATCC, Rockville, MD). Human mesothelioma cell lines SPC111, ZL5, ZL55, SPC212 and ZL34, were obtained from the University Hospital of Zurich (Switzerland) [50]. HeLa and HEK293T cells were maintained in DMEM medium supplemented with 10% FBS (Gibco, Basel, Switzerland) and 1% Penicillin/Streptomycin solution (1% PS; Gibco); all others in RPMI-1640 (Sigma-Aldrich, Buchs, Switzerland) containing 10% FBS supplemented with 2.5 μg/ml Amphotericin B (Corning, NY, USA). All cells were maintained at 37°C in a humidified 5% CO_2_ atmosphere.

### Lentiviral (LV) constructs, vector production and lentivirus isolation

For CR overexpression, pLV-CALB2 was used [23]; and for CR downregulation CALB2 shRNAs [7]. pLKO.1-sh*GFP* (plasmid #30323) was obtained from Addgene. Lentivirus particles were produced as described before [7]. Briefly, HEK293T cells were co-transfected by the CaPO_4_ method with 3 μg of the envelope plasmid pMD2.G-VSVG (Addgene plasmid #12259), 8 μg of the packaging plasmid psPAX2 (Addgene plasmid #12260) and 10 μg of the transfer plasmid. Lentivirus in the supernatant of HEK293T cells was harvested 48 h and 72 h after transfection. The supernatant was filtered (0.45 μm) and resuspended in DMEM containing 10% FBS and 1% PS solution. To stably express Renilla Luciferase in MSTO-211H cells the GFP cassette in pLVTHM was replaced with a cDNA coding for the Renilla luciferase pGL4.74[hRluc/TK] (Promega). Briefly, the plasmid was digested with *HindIII*, filled with Klenow enzyme, and then digested with *XbaI*. The fragment was inserted into the *PmeI* and *SpeI* sites of the backbone of pLVTHM to produce the final plasmid pLV-hRluc. Lentivirus were produced using the same envelope plasmid as above and the packaging plasmid pCMV-dR8.91 (kind gift from Prof. D. Trono, EPFL, Switzerland).

### LV titration by limiting dilution

Lentiviral particles at dilutions of 10^-3^ to 10^-7^ were used to infect HeLa cells in 6-well plates (50,000 cells/well). The medium was replaced after 48 h with a selection medium containing 2 μg/ml puromycin (Sigma). At day 12 post-infection cells were washed and stained with crystal violet, colonies were counted and lentiviral titer determined.

### Establishment of stably transduced cell lines

pLV-CALB2 lentiviral vectors were used to stably express CR in different cell lines. Cells were tested for CR expression by Western Blot analysis using the CR antibody CR 7699/4 (Swant, Marly, Switzerland). MSTO-211H cells were infected with a lentivirus carrying the hRluc reporter gene. After each passage, cells were lysed and the stable and long-lasting expression of Renilla luciferase was measured using Renilla Juice (p.j.k.-GmbH, Kleinblittersdorf, Germany) in a Turner Designs TD-20/20 Luminometer (Sunnyvale, CA, USA) according to the manufacturer’s protocol.

### Western blotting

Protein extracts (cleared lysates) were obtained from cells grown to 70% confluence using standard RIPA buffer (50 mM Tris, 150 mM NaCl, 0.1% sodium dodecyl sulfate (SDS), 0.5% sodium deoxycholate, 1% Triton X-100, pH 7.4) containing protease inhibitor cocktail (Quartett GmbH, Berlin, Germany) and 1 mM sodium orthovanadate (Na_3_V0_4_; Sigma). Cells were collected using a cell scraper, incubated on ice for 5-10 min and centrifuged at 12,000 x *g* for 20 min at 4°C. Proteins (40 μg) were separated by 10% SDS-PAGE, transferred onto nitrocellulose membranes [7], blocked with 5% BSA in TBS-Tween for 1 h and incubated overnight at 4°C with the following primary antibodies diluted in 2% BSA: rabbit polyclonal anti-Calretinin (1:10,000; CR 7699/4), rabbit polyclonal anti-FAK (1:1,000; Cell Signaling Technology, Danvers, MA, USA), rabbit polyclonal anti-p-FAK Tyr^397^ (1:1,000; Cell Signaling Technology), and rabbit polyclonal anti-GAPDH (1:5,000; Sigma); followed by incubation with secondary goat anti-rabbit or anti-mouse (HRP)-labeled antibodies (Sigma) at a dilution of 1:10,000. The signals were detected as described in [7]. For the EMT analysis membranes were incubated overnight at 4°C with a rabbit monoclonal anti-E-cadherin antibody (1:1,000; #3195; Cell Signaling Technology) and a mouse monoclonal N-cadherin (1:1,000; # 610920; BD Bioscience).

### Migration/proliferation and invasion assays

A ‘scratch wound assay’ was performed in both cases. Cells were grown to confluence in 96-well ImageLock plates (Essen Bioscience Inc., Ann Arbor, Michigan, USA) pre-coated with a thin layer of 0.1 mg/ml Matrigel Basement Membrane Matrix (Corning, Cat. No.354234). A scratch of about 1 mm width was created using the 96 well-plate woundmaker tool (Essen Bioscience) as described by the manufacturer. For the migration/proliferation assay, cells repopulating the scratch area grew directly on the surface of the ImageLock plates. In the case of the invasion assay, 50 μl of 1mg/ml of Matrigel Basement Membrane Matrix (10-times higher concentration as the layer on which cells initially grew; for details see Supplemental Methods) was added to each well after the scratch, and cells were incubated for 30 min. Finally 100 μl of medium was added on top of the cells and plates were scanned at a 2-h frequency using the Incucyte™ Live-cell Imaging System (Essen Bioscience). Images were evaluated with the IncuCyte™ software system. The Transwell invasion assay is described in detail in the Supplemental Methods.

### Immunofluorescence

Cells were seeded on 12-mm glass coverslips and fixed for 15 min with 4% paraformaldehyde when they had reached approximately 60-70% confluence, blocked with TBS-containing donkey serum (10%) and incubated overnight at 4°C with the following antibodies diluted in TBS 1X: goat polyclonal anti-Calretinin (1:500; cat#CG1, Swant) and anti-FAK (1:50; Cell Signaling). After washing, the cell-containing coverslips were incubated for 3 h at room temperature with the following secondary antibodies: Alexa Fluor 488-conjugated donkey anti-rabbit IgG (1:100; Jackson Immunoresearch Laboratories, West Grove, PA, USA) and Cy5-conjugated donkey anti-goat IgG (1:100; Jackson Immunoresearch Laboratories). Nuclear DNA was stained using DAPI (5 μg/ml; Molecular Probes, Eugene, OR) and coverslips were mounted with Hydromount solution (National Diagnostics, Atlanta, GA). Images were acquired using a LEICA fluorescent microscope DM6000B (Wetzlar, Germany) equipped with a Hamamatsu camera C4742-95 (Bridgewater, NJ).

### Subcellular fractionation and Western blotting

Nuclear and cytoplasmic protein fractions were prepared using the Nuclear Extraction KIT (#ab113474, Abcam) following the manufacturer’s instructions. Equal amounts (40 μg) were separated by 12.5% SDS-PAGE. Membranes were probed with anti-Calretinin (1:10,000; CR 7699/4), anti-FAK (1:1,000), rabbit monoclonal anti-Histone H3 (1:1,000; Cell Signaling Technology) and rabbit polyclonal anti-α-Tubulin (1:1,000; Cell Signaling Technology).

### Co-immunoprecipitation (Co-IP)

Cells (70–90% confluence) were lysed with 1 ml ice-cold lysis buffer (150 mM NaCl, 1% Triton X-100, 50 mM Tris HCl, pH 8.0) supplemented with protease and phosphatase inhibitors. Following 30 min incubation cells were centrifuged (10,000 × g for 10 min at 4°C). 50 μL of supernatant was aliquoted for total cell lysate analysis. To pull down calretinin, 2-4 μg of the anti-CR antiserum CR 7699/4 was added to the remaining supernatant. 100 μl of μMACS protein A MicroBeads (Miltenyi Biotec, Auburn, USA) were added to the lysate and incubated at 4°C for 30 min. Samples were loaded on MACS separation columns (Miltenyi Biotec) and subjected to magnetic immunoprecipitation. The columns were washed 3 times with a wash buffer (150 mM NaCl, 1% NP-40, 0.5% sodium deoxycholate, 0.1% SDS, 50 mM Tris HCl, pH 8.0) and protein complexes were eluted in 50 μL of pre-warmed SDS gel loading buffer 1X (50 mM Tris HCl, pH 6.8, 50 mM DTT, 1%SDS, 0.005% bromophenol blue, 10% glycerol), subjected to electrophoresis (SDS-PAGE) and subsequent Western Blotting. Normal rabbit polyclonal IgG (3 μl #12-370, Merck Millipore, Darmstadt, Germany) was used as a non-specific control for the co-IP experiments. Membranes were probed with anti-Calretinin (1:10,000; CR 7699/4), anti-FAK (1:1,000) and mouse monoclonal anti-Paxillin (1:2,000; BD Bioscience).

### FAK inhibitor treatment and viability assays (MTT)

3,000 cells/well were seeded in 96-well plates (TPP) and grown for 48 h. The FAK inhibitor Defactinib (VS-6063, PF-04554878, Selleckchem, Houston, USA) was solubilized in dimethyl sulfoxide (DMSO) and added in a concentration range from 2.5 to 10 μM. The MTT assay was performed after 48 h-72 h to determine the number of viable and proliferating cells [30]. The same concentration of DMSO without the inhibitor was used as a vehicle control.

### Pathway-specific PCR array

Total RNA was isolated using the RNeasy mini kit (Qiagen, Germany) and reverse-transcribed into single stranded cDNA using the RT^2^ First Strand Kit (Qiagen) following the manufacturer’s instructions. Differential expression of EMT genes was analyzed using a RT^2^ profiler PCR array specific for EMT Pathway (PAHS-090ZR-12, Qiagen). Quantitative real-time PCRs (qPCR) were performed in a DNA thermal cycler (Corbett Rotor gene 6000, QIAGEN Instruments AG, Hombrechtikon, Switzerland). The following thermal profile was applied: 1 cycle at 95 °C for 10 min, 40 cycles at 95 °C for 15 s and 60 °C for 30 s. Differences in fold expression were calculated following the 2^-ΔΔCt^ method [51].

### Downregulation of CR and GFP expression in MSTO-211H cells using lentiviral-mediated shRNA *in vitro*

MSTO-211H cells (25,000/well) were seeded into 6-well plates and grown for 24 h. LV containing a *CALB2* or a *GFP* shRNA were added with a multiplicity of infection (MOI) of 10. 72 h later cells were collected and 30 μg of protein extract was subjected to 10% SDS-PAGE and subsequent Western Blotting.

### Downregulation of CR and GFP expression in MSTO in an orthotopic mouse model

NOD SCID gamma (NSG) mice were utilized (4 mice per group). All experiments were performed with permission of the local animal care committee (Canton of Fribourg, Switzerland) and according to the present Swiss law and the European Communities Council Directive of 24 November 1986 (86/609/EEC). Briefly, MSTO-211H-Rluc cells were transduced for 24 h with a lentiviral vector containing either an shRNA against *GFP* (control group) or against *CALB2* (test group) at a MOI of 10. Mice were separated into two groups and injected with cells pre-treated with shGFP or with shCALB2. Direct intraperitoneal (i.p.) injection of 1.5x10^6^ tumor cells in 200 μl PBS serum-free media was performed and mice were scanned at days 16 and 30 post-injection in order to follow tumor progression with the IVIS Lumina II *In Vivo* Imaging System (Caliper Life Sciences, Hopkinton, USA). For the Renilla luciferase detection imaging, 1 mg/kg ViviRen™ *In Vivo* Renilla Luciferase Substrate (Cat#P1231; Promega, Dübendorf, Switzerland) was injected i.p. before imaging. Images were acquired for 5-30 seconds depending on signal strength. Luminescence of the tumor was quantitatively evaluated using the Living Image 4.2 Software and the BLI signal was reported as photons/s/cm^2^/sr. Organ samples were collected (diaphragm and parietal layer) for histological analysis.

### Immunohistochemistry

Sections (3 μm) were de-paraffinized and treated with Tris/EDTA (1 mM/0.1 mM, pH 9) for the antigen retrieval by heating the sections in boiling water for 20 min, followed by 20 min incubation with 0.3% hydrogen peroxide. Then tissue was permeabilized with 0.1% PBS-Tween and blocked with PBS containing 2% BSA and 1% bovine serum for 1 h. Sections were incubated with a primary antibody rabbit polyclonal anti-Calretinin (1:500; CR 7699/4) or a mouse monoclonal anti-FAK antibody (1:200; clone 4.47, Merck Millipore) overnight at 4°C. The next day, sections were incubated with a secondary biotinylated antibody (1:200) at room temperature for 2 h and with VECTASTAIN® Elite ABC reagent (Vector Laboratories, Servion, Switzerland) for another 3 h. DAB (Sigma) staining was followed by hematoxylin counterstaining. Slides were scanned using a whole-slide imaging system from Hamamatsu (Nanozoomer, 2.0-HT).

### Statistical analysis

MTT results from at least 3 independent experiments were pooled together; each sample was measured in triplicates. Mean and standard deviation are shown in the figures. The statistical significance of all the experiments was calculated with the GraphPad Prism software (GraphPad Software Inc., San Diego, CA, USA). A two way-ANOVA was used to analyze the FAK inhibitor (factors: dose and CR expression) and the orthotopic mouse model experiments (factors: treatment shCALB2/shGFP and time). In both cases a Sidak’s multiple comparison was used as post-hoc test. A one sample t-test (two-sided) was performed to quantify the protein levels of nuclear FAK and p-FAK (Tyr^397^), and for the comparison of the wound closure times. Differences with p-values of less than 0.05 were considered significant.

## SUPPLEMENTARY MATERIALS FIGURES AND VIDEOS











## Abbreviations

CR: calretinin
FAK: focal adhesion kinase
MM: malignant mesothelioma
EMT: epithelial-to-mesenchymal transition
ECM: extracellular matrix
BLI: bioluminescent imaging
