# Supplementary material for: Calretinin promotes invasiveness and EMT in malignant mesothelioma cells involving the activation of the FAK signaling pathway
Source: Oncotarget. 2018 Nov 20;9(91):36256–72. doi: 10.18632/oncotarget.26332 (PMC6284738; doi:10.18632/oncotarget.26332)
Supplement: Supplementary file 1 [file oncotarget-09-36256-s001.pdf]

Calretinin promotes invasiveness and EMT in malignant mesothelioma cells involving the activation of the FAK signaling pathway

SUPPLEMENTARY MATERIALS

SUPPLEMENTARY METHODS

Transwell (Boyden chamber) invasion assay

The invasive ability of MM cells was additionally investigated using Fluoroblock transwell inserts (8-µm pore size; Corning, Chemie Brunschwig AG, Switzerland). The inserts were coated with 100 µl of Matrigel Basement Membrane Matrix at a final concentration of 200-300 µg/ml and incubated 2 h at 37°C for solidification. Un-coated inserts were used to assess migration. Briefly, serum-starved cells (SPC111 and SPC111-CR) were seeded in the upper chamber of the inserts (50,000 cells/insert) and medium-containing 10% FBS was added to each of the basal chambers. Plates were incubated for 20-22 h at 37°C in a 5% CO_2-humidified incubator. Then the medium was carefully removed from the apical chambers and non-migrating or non-invading cells on the upper surface of the filter were removed with cotton swabs. Cells that migrated or invaded to the underside of the membrane inserts were loaded with Calcein-AM (4 µg/ml; Invitrogen, Zug, Switzerland) in HBSS (Gibco) and incubated for 1 h. The fluorescence of migrating and invading cells was measured on a fluorescent plate reader at 517 nm. The relative fluorescent units (RFU) represent the percentage of invasion of the cells, and are calculated as the mean RFU of invading cells/mean of RFU of migrating cells. Experiments were performed in triplicates and an unpaired t-test was used to determine statistical significance of the results.

Scratch assay to measure proliferation/migration and invasion

MM cells (red) were grown on a thin layer of Matrigel (0.1 mg/ml; light blue). After the scratch, cells were grown in medium (pink) in the proliferation/migration assay. For the invasion assay, cells and the scratch region was covered by Matrigel (1 mg/ml; dark blue) and medium was added on top. In the invasion assay cells need to cross the Basement Membrane Matrix.

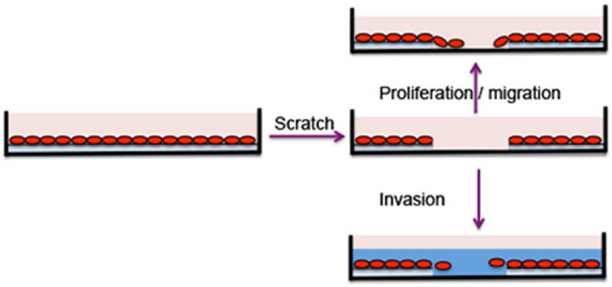

REFERENCES

1. Blum W, Schwaller B. Calretinin is essential for mesothelioma cell growth/survival *in vitro*: a potential new target for malignant mesothelioma therapy? Int J Cancer. 2013; 133:2077–88. https://doi.org/10.1002/ijc.28218

2. Brown MC, Perrotta JA, Turner CE. Identification of LIM3 as the principal determinant of paxillin focal adhesion localization and characterization of a novel motif on paxillin directing vinculin and focal adhesion kinase binding. J Cell Biol. 1996; 135:1109–23. https://doi.org/10.1083/jcb.135.4.1109

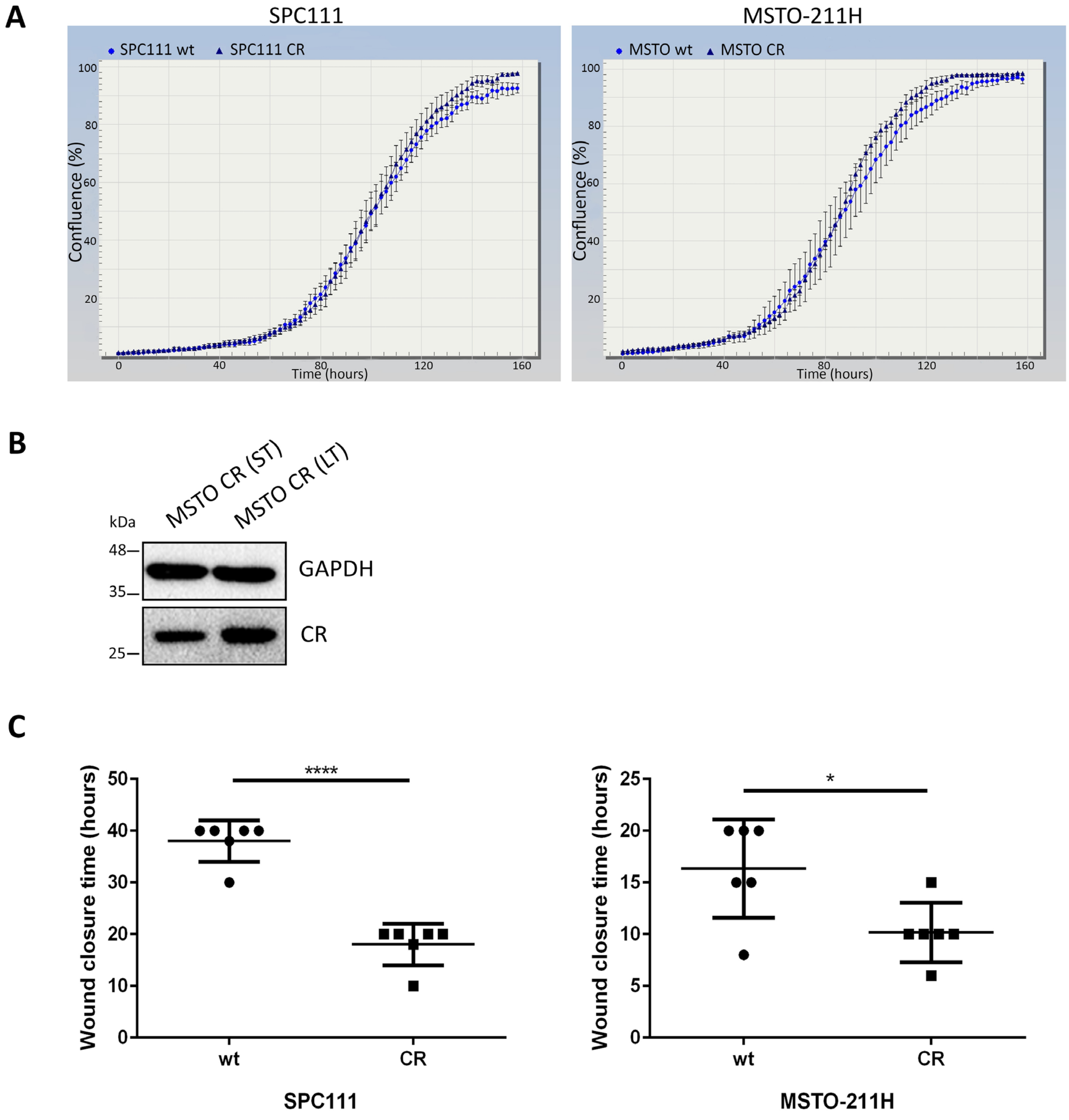

Supplementary Figure 1: Comparison of growth curves, CR protein levels and wound closure times of wt and CR-overexpressing MM cells. **(A)** No differences are observed in the growth curves between wt and CR-expressing cells (SPC111, left graph; MSTO-211H, right graph) at passages >5. **(B)** CR protein levels show no significant changes between short-term (ST <10) and long-term (LT >30) passages in MSTO-CR cells. A slight trend towards higher CR levels at later passages was observed. **(C)** Wound closure times were quantified in CR-overexpressing and wt cells. SPC111-CR cells showed an average wound closure time of 18 h, while wt cells showed an average time of 38 h ($^{****}p \leq 0.0001$). The corresponding values in MSTO-211H-CR and wt cells were 10 h and 16 h, respectively ($^{*}p \leq 0.05$) (n = 6 independent experiments).

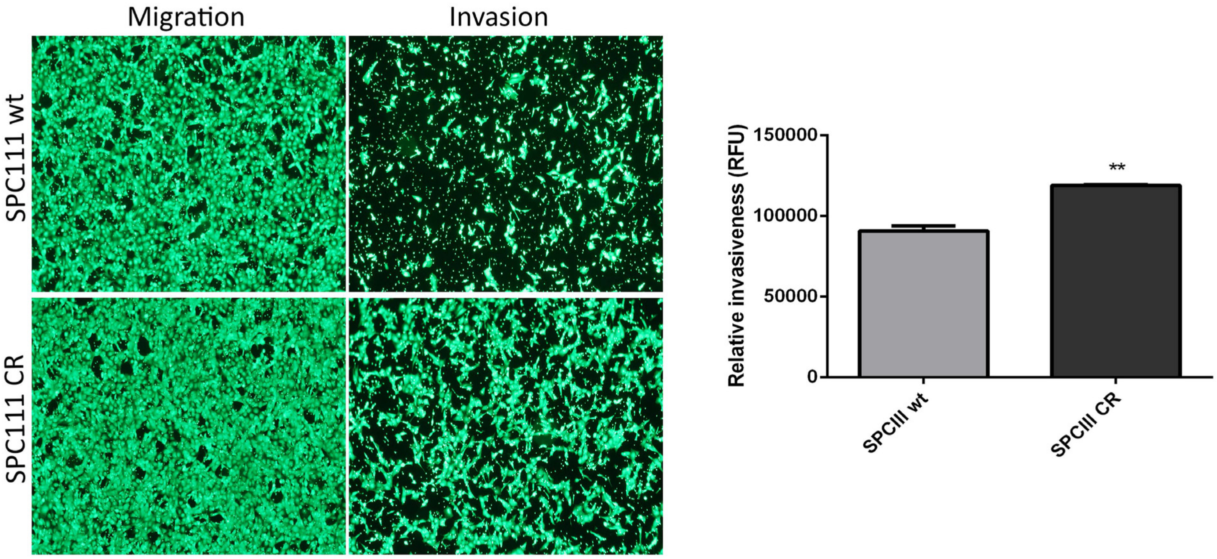

Supplementary Figure 2: Invasion assay measured with Fluoroblock transwell inserts in SPC111 cells. SPC111-CR cells showed a significant enhanced invasive phenotype compared to the corresponding SPC111 wt cells ($^{**}p \leq 0.01$; n = 3 independent experiments).

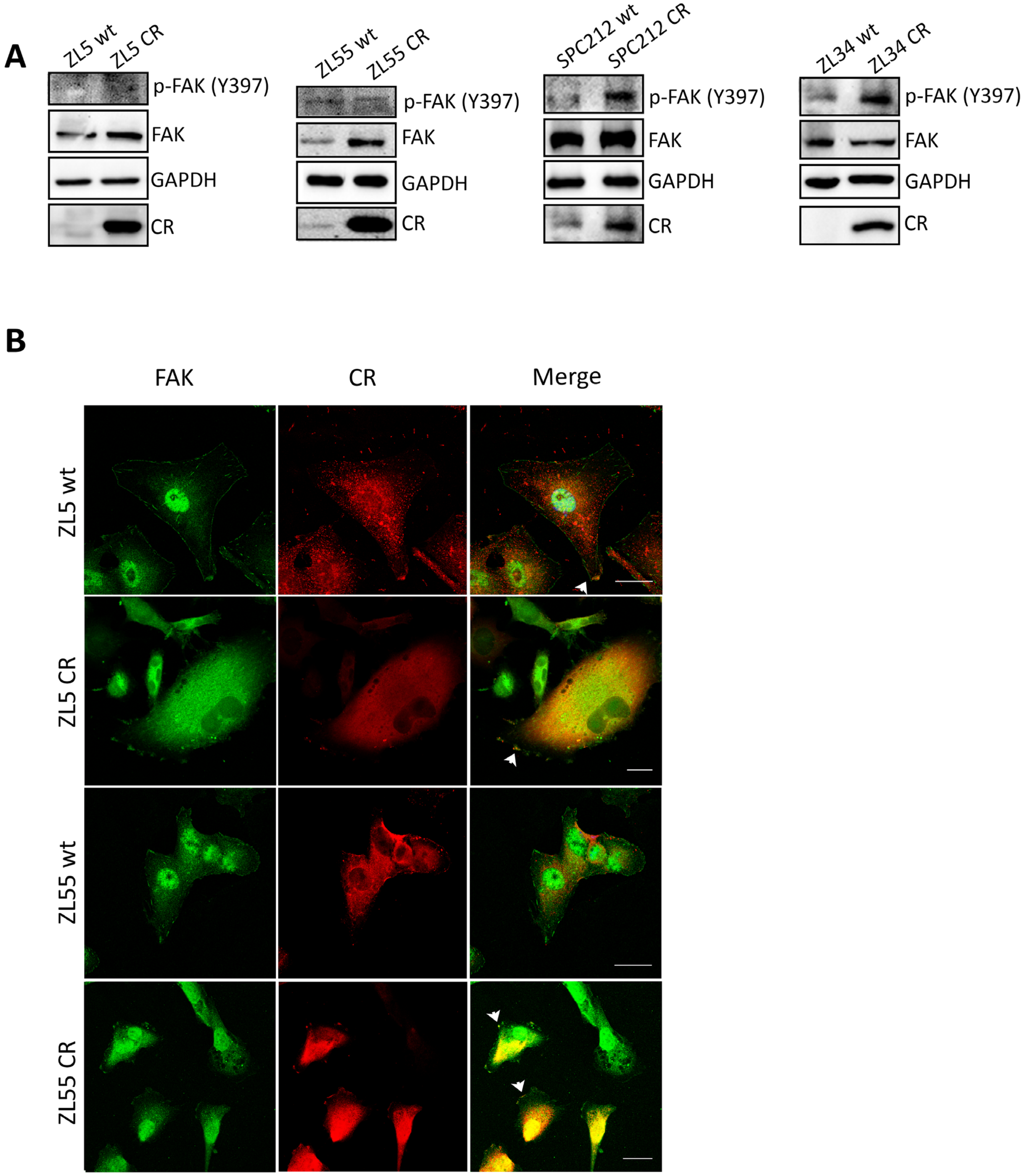

Supplementary Figure 3: FAK protein levels and immunofluorescence analyses in other CR-overexpressing MM cell lines. (A) CR overexpression led to an up-regulation of FAK levels in the cell lines ZL5 and ZL55. In addition, p-FAK (Tyr^{397}) levels were increased in ZL34-CR and SPC212-CR cells. GAPDH was used as loading control. **(B)** Representative confocal images from fixed cells of FAK (green) and CR (red) in wt and CR-overexpressing cells from epithelioid origin, ZL5 and ZL55. FAK levels were clearly increased in CR-expressing cells when compared to the corresponding wt cells. In addition, both CR-overexpressing cell lines showed CR co-localization with FAK (represented by the yellow color in the merged image) at the leading edge of the cells forming punctate-like patterns along the plasma membrane (arrowheads); while in wt cells this co-localization was rare. Scale bars: 20 µm in images ZL5 CR, ZL55 CR; 10 µm in images ZL5 wt, ZL55 wt. For more details of basal CR protein levels in the different MM cell lines, see [1].

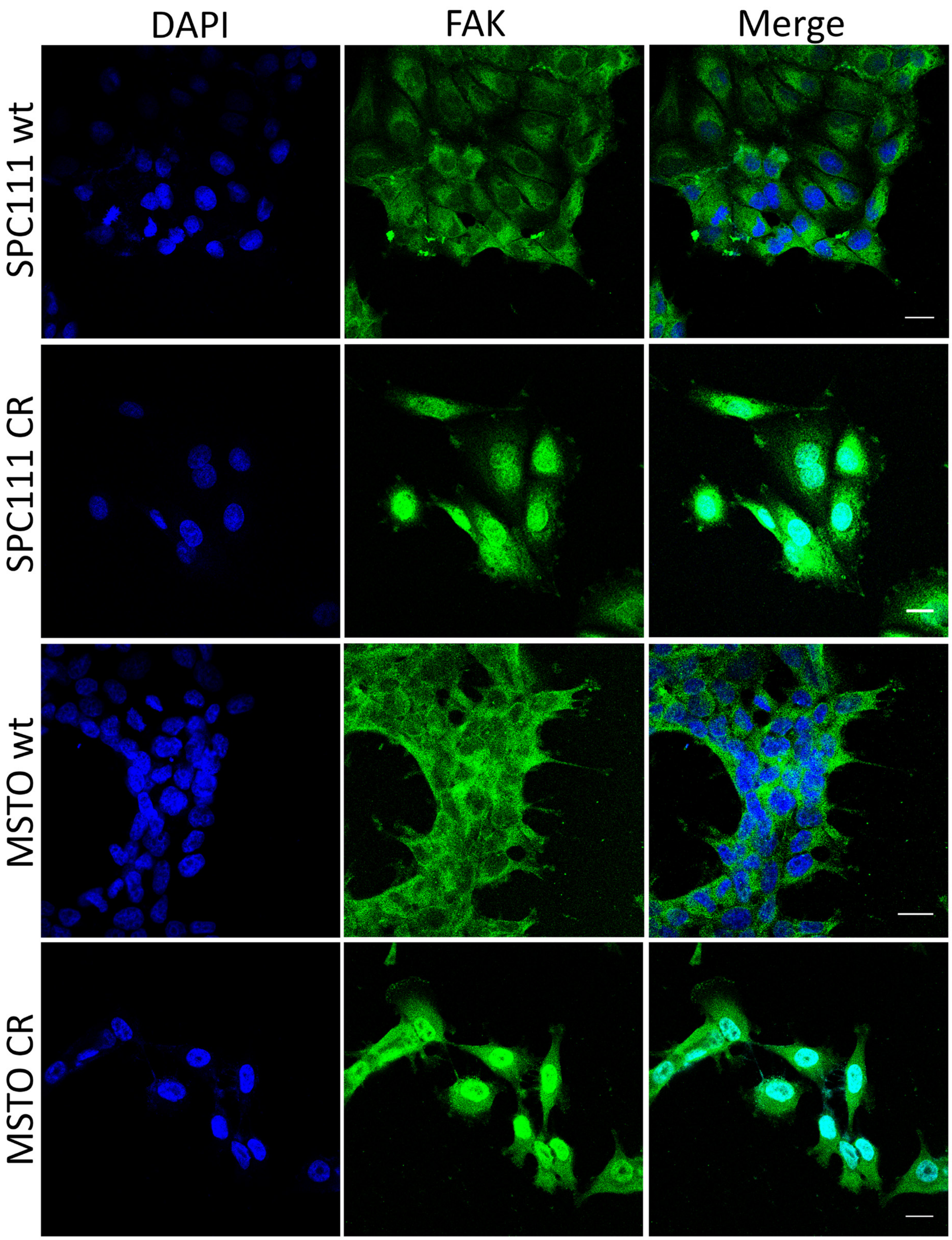

Supplementary Figure 4: FAK immunofluorescence and DAPI staining of SPC111 and MSTO-211H wt and CR-overexpressing cells. Representative confocal fluorescence images from fixed cells stained for FAK (green) and with DAPI (blue). In both CR-overexpressing cell lines FAK showed a strong nuclear staining that co-localized with DAPI (evidenced by the light blue (cyan) color of the in the merged image). Scale bar: 20 µm.

**Paxillin LD2**	LDRLLLELNAVQHNP
**CR EF-hand 5**	LDALLGDLYEKNKKE

Supplementary Figure 5: Sequence comparison of the LD2 domain of paxillin and the F-helix of EF-hand 5 of calretinin. The conserved “LD domain” residues are boxed in yellow. The previously identified FAT-domain of FAK interacting with paxillin consists of an aspartate (D; red) and the residues LLLQ all aligned on the same side of an alpha helix (for details, see [2]). Note the arrangement of the essential amino acids (red and green) is almost identical in CR; the only difference is a change from asparagine (Q) to glutamine (N) in the sequence of CR.

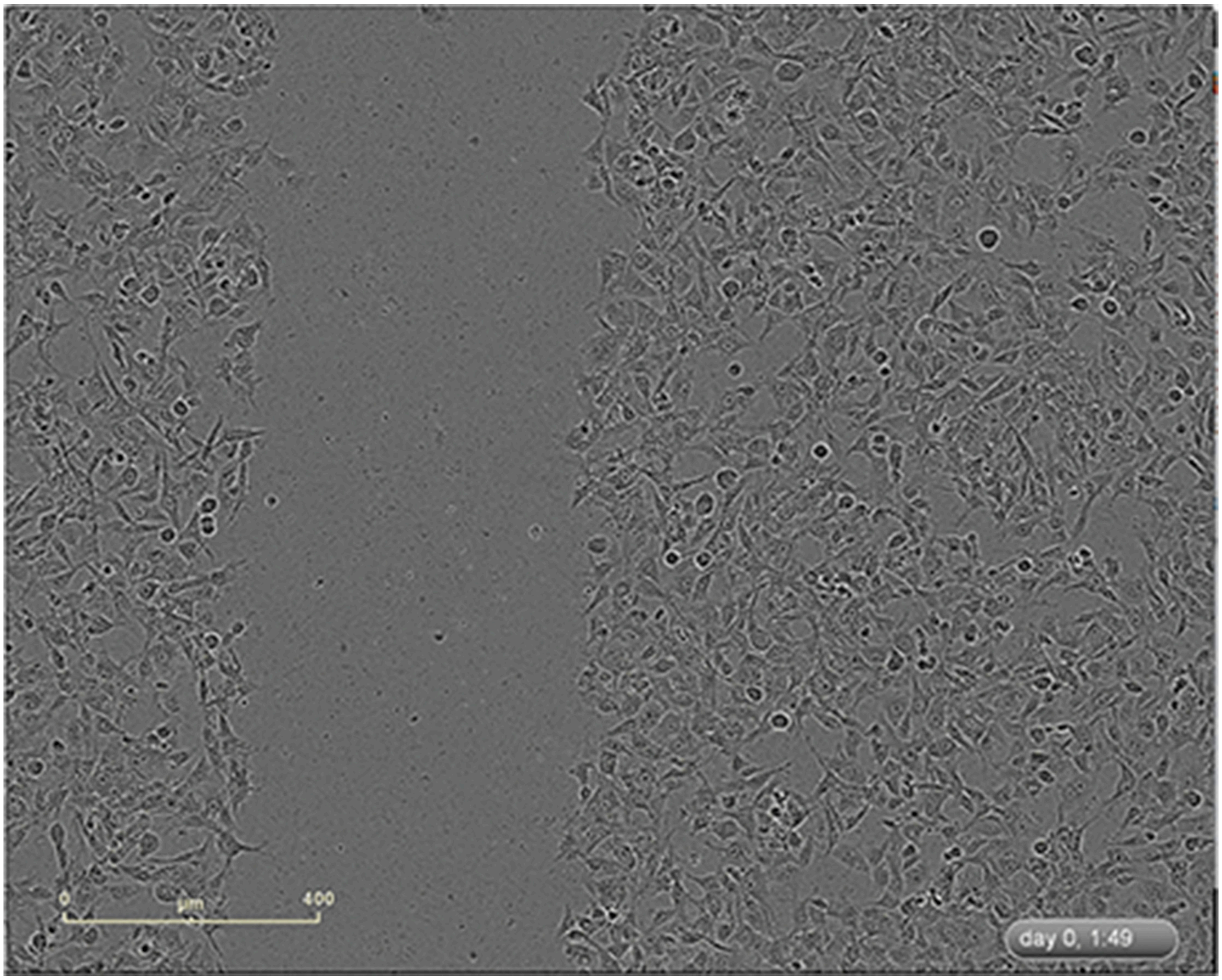

Supplementary Movie 1: Time-lapse video of invasion assay using MSTO-211H-CR cells.

See Supplementary File 1

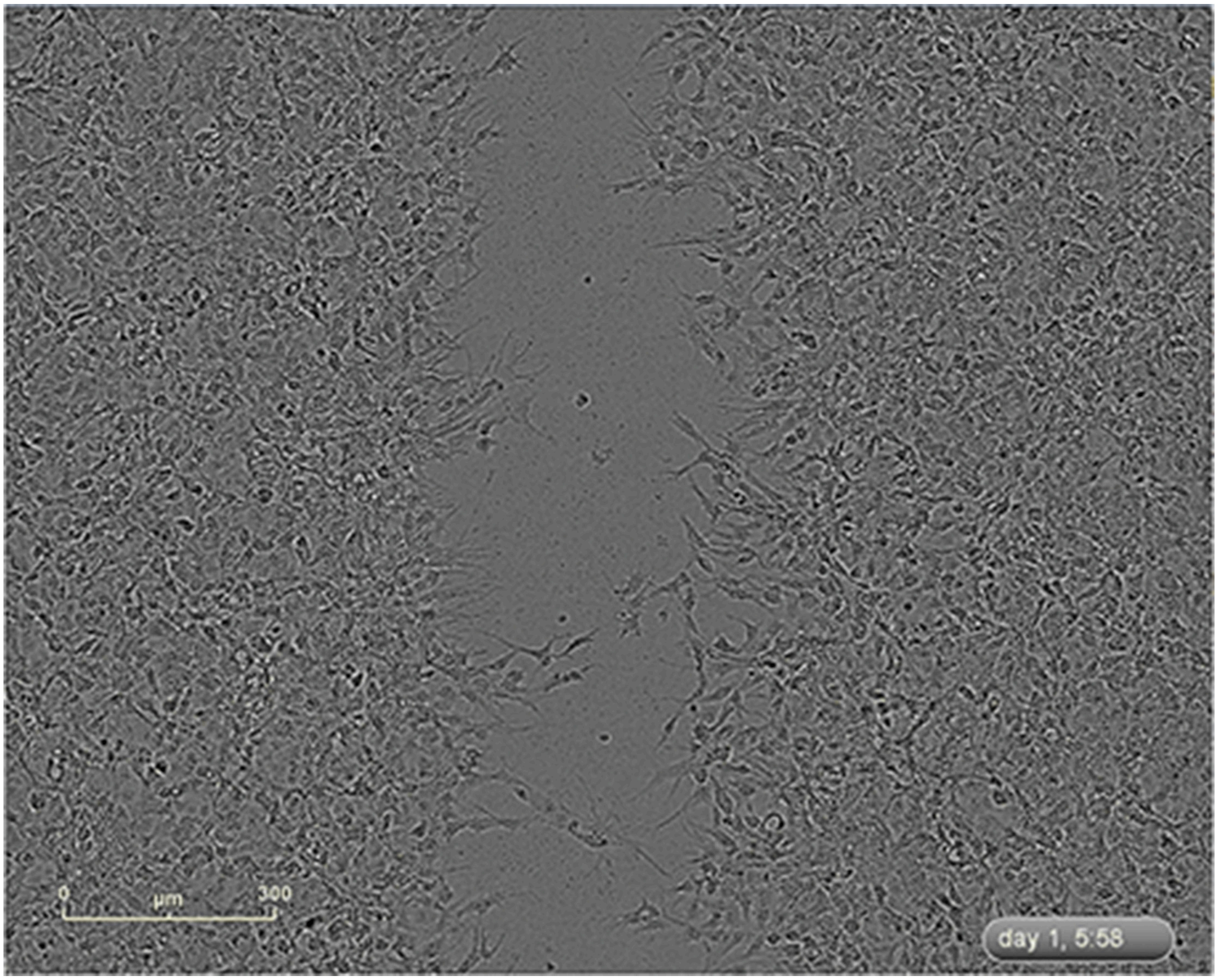

Supplementary Movie 2: Time-lapse video of invasion assay using MSTO-211H wt cells.

See Supplementary File 2

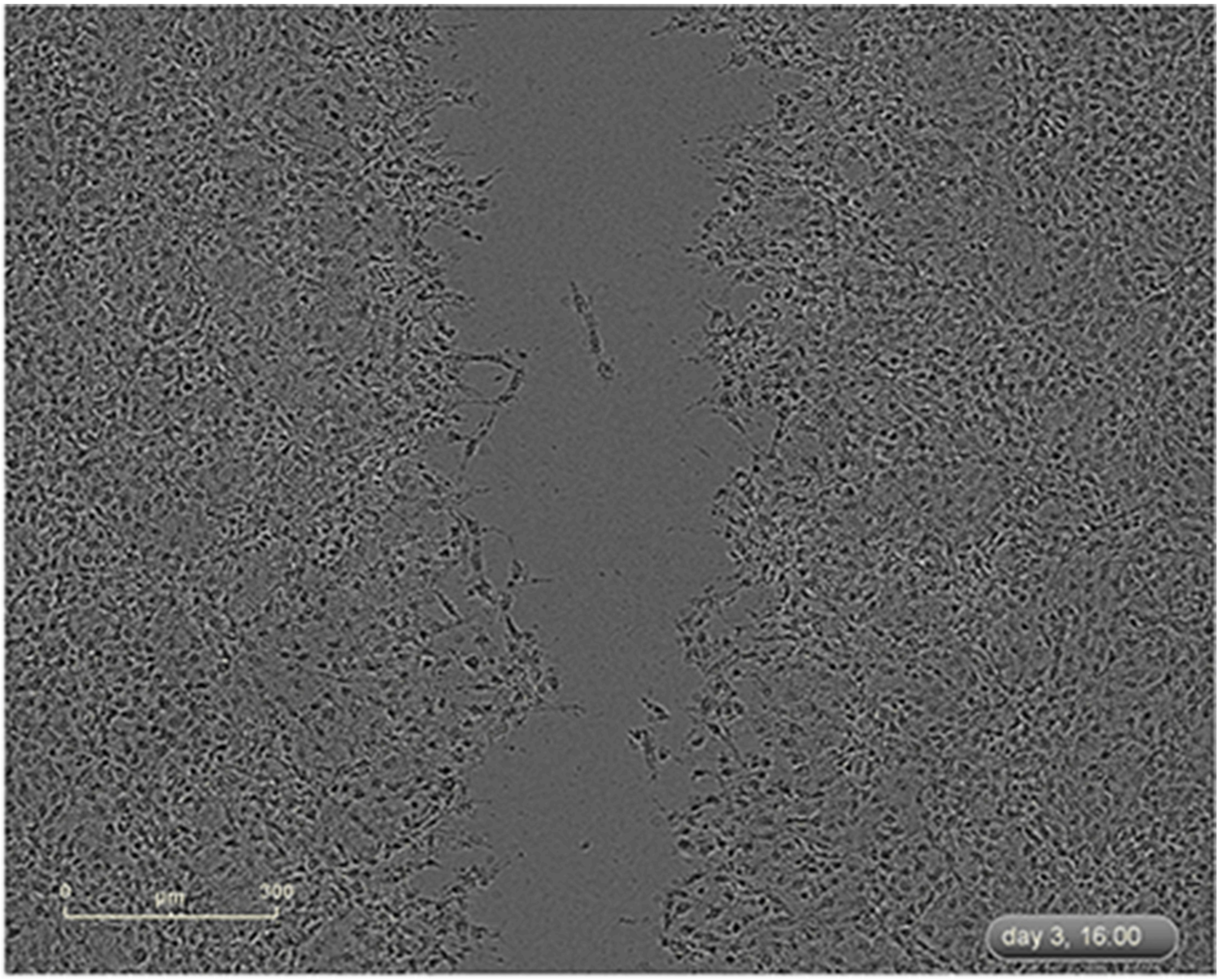

Supplementary Movie 3: Time-lapse video of invasion assay using SPC111 wt cells.

See Supplementary File 3

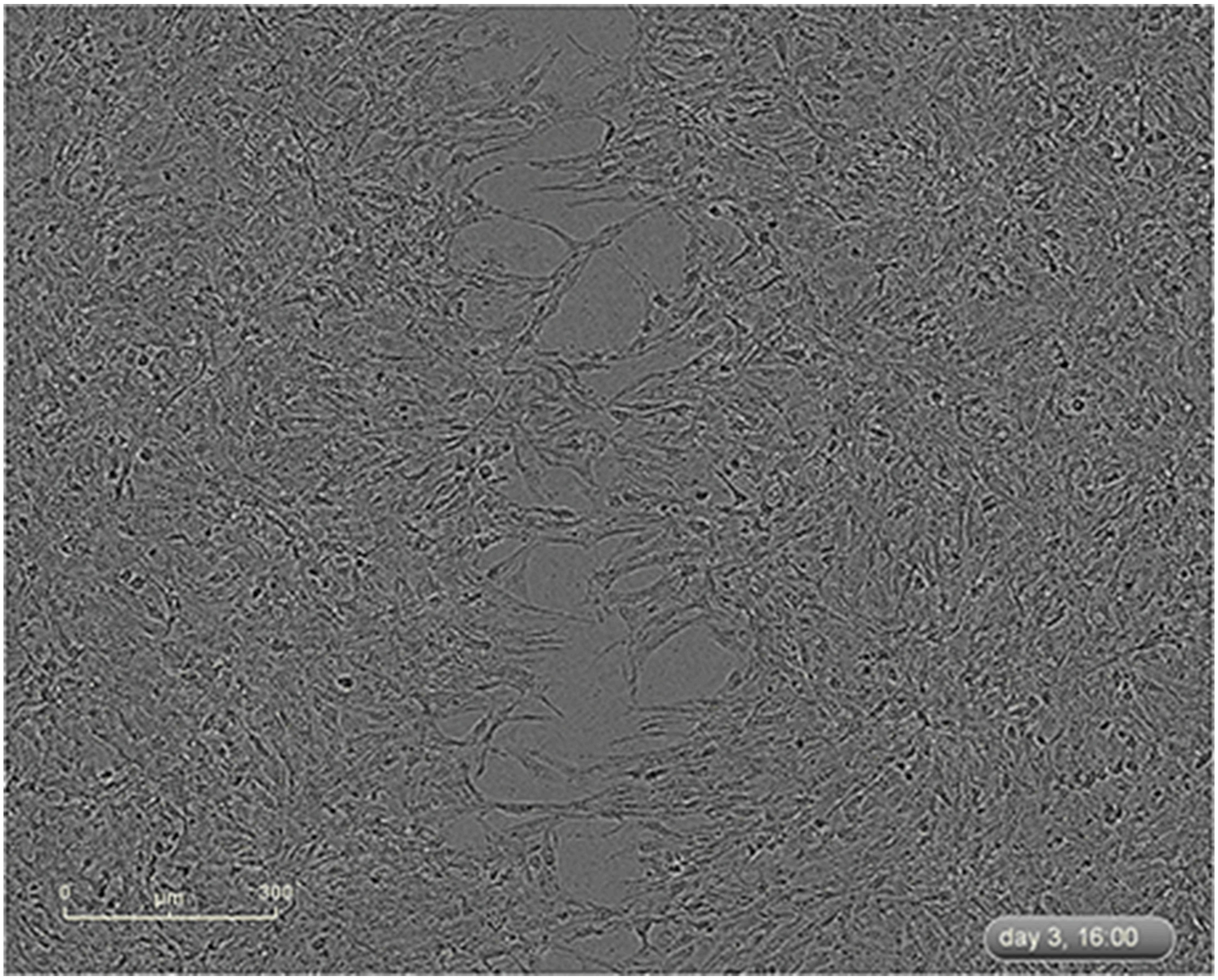

Supplementary Movie 4: Time-lapse video of invasion assay using SPC111-CR cells.

See Supplementary File 4
